# Trends in the Prevalence of Obesity Among U.S. Active Component Service Members and Civilians, 2013–2023

**Published:** 2026-02-04

**Authors:** Samuel D. Emmerich, Sithembile L. Mabila, Bryan Stierman, Cynthia L. Ogden

**Affiliations:** Epidemic Intelligence Service, U.S. Centers for Disease Control and Prevention, Atlanta, GA: MAJ Emmerich; National Center for Health Statistics, Centers for Disease Control and Prevention, Hyattsville, MD: MAJ Emmerich, Dr. Stierman, Dr. Ogden; Epidemiology and Analysis Branch, Armed Forces Health Surveillance Division, Public Health Directorate, Defense Health Agency, Silver Spring, MD: Dr. Mabila

## Abstract

Trends in obesity among U.S. active component service members (ACSMs) and civilians are relevant to military recruitment and retention, as excess body weight is a common disqualification for military service. This study utilized measured height and weight data from the Military Health System Data Repository for ACSMs (cumulative n=12,262,745) and the National Health and Nutrition Examination Survey for civilians aged 17-62 years (cumulative n=19,334). Accounting for the design of each data source, the prevalence of obesity (body mass index >= 30 kg/m ^2^ ) and body mass index (BMI) distributions were calculated. Joinpoint software and polynomial regression were used to assess trends over time. From 2013 through 2023, obesity prevalence increased among ACSMs, from 14.7% to 24.2%. Although obesity rates among civilians were consistently higher, this gap narrowed over the course of the decade. The same pattern was seen in young men (ages 17-24 years). Civilians have greater proportions than ACSMs within the highest classes of BMI. Persistently high obesity prevalence among ACSMs overall and in young men, particularly since 2019, may affect military recruitment, retention, and ultimately, strength and readiness.

**What are the new findings?**

From 2013 through 2023, the prevalence of obesity increased significantly among U.S. active component service members, 2019 to 2023 in particular, while prevalence among civilians remained consistently high. The pattern of obesity is especially relevant in young men, the largest source of potential and newly accessed military recruits.

**What is the impact on readiness and force health protection?**

The persistently high prevalence of obesity among civilians and growing prevalence of obesity among active component service members in general, and among young men in particular, may affect military recruitment, retention, and ultimately, strength and readiness.

The U.S. Department of Defense (DOD) experienced agency-wide recruitment shortfalls in 2022 and 2023. ^1^ Excess body weight is a common disqualification for recruitment and retention of military members. ^2^ Some authors have suggested that the high prevalence of weight-ineligible young people has compromised national security by reducing recruitment. ^3^ Obesity also places a substantial burden on the Military Health System (MHS). ^4^

Reports have found that the prevalence of obesity in U.S. military members increased slightly during the COVID-19 pandemic, ^5^ but prevalence of obesity in the overall U.S. civilian adult population remained level. ^6^ Examining whether trends in obesity prevalence are similar, when sex and age standardized, for the active component military and civilian populations, as well as for young men from both populations, is ultimately relevant to U.S. military strength and readiness.

The objective of this study was to examine trends over the past decade in the prevalence of obesity among U.S. active component service members (ACSMs) and civilians ages 17-62 years, both overall and by sex, to understand whether trends in these populations were similar or different. This study highlights trends in young men ages 17-24 years among both populations, to examine differences in the prevalence of obesity between potential civilian and newly accessed military recruits. Finally, this study visualizes the cross-sectional distribution of body mass index (BMI) at the end of the study period, in both men and women, to compare the age-standardized distribution of BMI categories between military and civilian populations.

## Methods

### Data sources

For the ACSM population, this study employed a census of medical records with measured height and weight data from January 1, 2013 through December 31, 2023 from the MHS Data Repository (MDR). An encounter record in MDR can be initiated by individuals seeking care or by a healthy individuals completing an annual physical examination requirement. For each calendar year (e.g., 2013, 2014, etc.), the first encounter that included a non-pregnant height and weight measurement for an individual was abstracted from the MDR and linked to demographic data from the Defense Medical Surveillance System (DMSS). ^7^ The same individual could be represented in multiple years of this study period, but never more than once every given year. Records with missing racial or ethnic group or sex data were excluded (n=271,679, 2.2%).

For civilians, this study utilized measured height and weight as well as demographic data from 4 survey cycles of the National Health and Nutrition Examination Survey (NHANES): 2013-2014, 2015-2016, 2017-March 2020, and August 2021-August 2023. NHANES is a cross-sectional, interview- and examination-based survey representative of the U.S. civilian, non-institutionalized population, approved by the National Center for Health Statistics (NCHS) Ethics Review Board. ^8^ Non-pregnant NHANES participants ages 17-62 years (i.e., ACSM age range) with measured height and weight were included in this study.

### Body mass index categories

BMI was calculated as weight in kilograms divided by height in meters squared, rounded to 1 decimal place. BMI categories were defined as underweight (BMI<18.5), normal weight (BMI 18.5<25.0), overweight (BMI 25.0<30.0), and obesity (BMI>=30.0). Obesity was further classified as class 1 obesity (BMI 30.0<35.0), class 2 obesity (BMI 35.0<40.0), and class 3 obesity (BMI>=40.0). ^9^ Records from ACSMs with BMI less than or equal to 12 or greater than or equal to 50 were considered implausible and excluded from this study (n=6,562, 0.1%).

### Statistical analysis

Analyses were conducted using R version 4.4.0 including survey package version 4.4-2 (R Foundation), SAS-Enterprise Guide version 8.3 (SAS Institute, Inc.), and Joinpoint Regression Program version 5.4.0 (National Cancer Institute). A 2-sided *p* -value of less than .05 was used to determine statistical significance.

### Prevalence of obesity

The crude prevalence of obesity among ACSMs was calculated both overall and by sex, age, racial and ethnic group, and branch of military service, for each year, 2013–2023. Because ACSM data are a census of the population, confidence intervals (CIs) were not calculated. Overall, and for every demographic group, the percentage point change and relative percentage change over the study period were calculated using the prevalence of obesity in 2013 and 2023.

For civilians, examination survey weights were used to estimate the crude prevalence of obesity overall and by sex, age, and racial and ethnic group, for each survey cycle; Korn and Graubard CIs were calculated, and estimates were evaluated for reliability according to the NCHS Data Presentation Standards for Proportions. ^10^ Percentage point change and relative percentage change were not calculated for civilians due to the unequal lengths and spacing of NHANES survey cycles.

Overall prevalence of obesity for ACSMs and civilians were also standardized to the sex and age structure of the ACSM study population in 2023 to account for demographic composition differences between and within these populations over time.

### Trends in obesity

Statistical testing for trends in obesity over time were conducted by sex, age, racial and ethnic group, and branch of military service to provide subgroup information, which is relevant for military retention and recruitment, particularly for young men (ages 17-24 years).

For ACSMs, Joinpoint software (using default settings and weighted BIC model) was used to identify inflection points in obesity prevalence over time, and to test whether apparent changes in slope at inflection points were significant. The difference in slope of trends before and after significant inflection points, measured in annual percentage point change, were reported.

For civilians, quadratic and linear trends in obesity prevalence over time were examined in regression models with the survey cycle modeled as an orthogonal polynomial, accounting for the unequal spacings and lengths of NHANES survey cycles, using the NCHS Guidelines for Analysis of Trends. ^11^ Because only 4 NHANES survey cycles were included in this study, Joinpoint software was not used to analyze civilian trends.

### Distributions of body mass index

The prevalence of each BMI-defined weight category was calculated and used to visualize the 2023 distributions of BMI by sex among ACSMs in 2023 and the civilian population from August 2021 through August 2023, standardized to the age structure of the ACSM study population in 2023.

## Results

### Demographics of active component service members and civilians

This study included a cumulative total of 12,262,745 ACSM records of measured height and weight from the MHS Data Repository from 2013 through 2023 **(Table 1)** . The demographic distribution of this study's ACSM population is similar to active duty members in the DOD 2023 Demographics Report. ^12^

**TABLE 1.** Demographics, U.S. Active Component Service Members and Civilians, Ages 17–62 Years, 2013–2023 ^a^

		Service Members ^b^		Civilians ^c^	
		No.	Proportion (%)	No.	Weighted Proportion (%)
Total	12,262,745	100	19,334	100
Sex				
Men	10,171,801	82.9	9,265	50.0
Women	2,090,944	17.1	10,069	50.0
Age, *y*				
17–24	4,950,752	40.4	3,673	16.9
25–34	4,624,351	37.7	3,906	22.4
35–44	2,193,378	17.9	3,994	21.3
45–62	494,264	4.0	7,761	39.4
Sex, age group, *y*				
Men, 17–24	4,014,865	32.7	1,881	8.8
Men, 25–34	3,858,480	31.5	1,877	11.5
Men, 35–44	1,876,631	15.3	1,816	10.4
Men, 45–62	421,825	3.4	3,691	19.2
Women, 17–24	935,887	7.6	1,792	8.1
Women, 25–34	765,871	6.2	2,029	10.9
Women, 35–44	316,747	2.6	2,178	10.8
Women, 45–62	72,439	0.6	4,070	20.2
Race and ethnicity				
Hispanic	1,942,831	15.8	4,898	18.2
Black, non-Hispanic	2,064,534	16.8	4,192	12.0
White, non-Hispanic	7,038,493	57.4	6,998	59.1
Other	1,216,887	9.9	3,246	10.7
Service branch				
Army	4,792,228	39.1		
Navy	2,693,611	22.0		
Air Force	2,975,221	24.3		
Marine Corps	1,544,593	12.6		
Coast Guard	130,272	1.1		
Other ^d^	126,820	1.0		

Abbreviations: No., number (unweighted); *y* , years.

a Data sources: Defense Medical Surveillance System (DMSS), 2013-2023; Military Health System Data Repository (MDR), 2013-2023; National Health and Nutrition Examination Survey (NHANES), 2013-2014 to Aug. 2021-Aug. 2023.

b Service member subgroup sample size ranges: men, 755,426–1,034,507; women, 181,172–201,182; ages 17-24 *y* , 347,540–501,015; 25-34 *y* , 362,316–481,673; 35-44 *y* , 177,713–222,560; 45-62 *y* , 38,787–49,178; Hispanic, 157,466–196,642; non-Hispanic Black, 160,731–204,752; non-Hispanic White, 495,575–756,582; other, 101,004–119,304; Army, 332,730–510,326; Navy, 180,424–268,924; Air Force, 227,918–312,870; Marine Corps, 100,761–162,609; Coast Guard, 6,831–25,797; other service, 856–48,645.

c Civilian subgroup sample size ranges: men, 1,854–3,155; women, 2,215–3,340; ages 17–24 *y* , 684–1,254; 25–34 *y* , 798–1,273; 35–44 *y* , 851–1,273; 45–62 *y* , 1,656–1,736; Hispanic, 879–1,565; non-Hispanic Black, 544–1,748; non-Hispanic White, 1,254–2,111; other, 535–1,254.

d Includes Space Force, Navy afloat, National Oceanic and Atmospheric Administration, Public Health Service, Office of Secretary of Defense, other / unknown.

This study included a cumulative total of 19,334 civilian participants from 4 survey cycles of NHANES **(Table 1)** . NHANES estimates are representative of the U.S. non-institutional, civilian population. ^13^

The population of ACSMs is younger (78.1% ages 17-34 years), with a higher percentage of men (82.9%) than the U.S. civilian population (39.3% ages 17-34 years, 50.0% men).

### Obesity trends overall

Sex- and age-standardized prevalence of obesity in ACSMs increased from 14.7% in 2013 to 18.7% in 2020; from the joinpoint at 2020, obesity prevalence rose more rapidly, to 24.2% in 2023 (difference in slope before and after joinpoint 1.33, *p*<0.001) **(Table 2)** . The standardized estimated prevalence of obesity among civilians, which was consistently higher than ACSMs throughout this period, increased from 31.3% (95% CI 29.2, 33.6) in the NHANES 2013-2014 survey cycle to 37.8% (95% CI 34.7, 40.9) 2017–March 2020 and then declined to 33.0% (95% CI 30.1, 36.0) August 2021–August 2023 (quadratic trend *p* =0.04, linear trend *p* =0.49) **(Table 3)** .

**TABLE 2.** Trends in Prevalence of Obesity ^a^ , U.S. Active Component Service Members, 2013–2023 ^b^

Year	2013	2014	2015	2016	2017	2018	2019	2020	2021	2022	2023	Percentage Point Change	Relative % Change
Total, *n*	1,232,019	1,216,338	1,178,812	1,165,274	1,170,057	1,169,221	1,174,685	1,016,301	1,023,155	980,285	936,598		
	%	%	%	%	%	%	%	%	%	%	%		
Standardized ^c^	14.7	14.7	15.2	16.1	16.7	17.3	17.8	18.7 ^c^	20.9	22.6	24.2	9.5	64.9
Crude	14.5	14.6	15.0	15.8	16.2	16.6	17.0 ^d^	18.0	20.5	22.4	24.2	9.6	66.2
Sex													
Men	15.7	15.7	16.2	16.9	17.3	17.7	18.1 ^d^	19.1	21.6	23.5	25.3	9.5	60.6
Women	8.3	8.3	8.8	9.6	10.4	11.1	12.0 ^d^	13.1	15.5	17.7	19.6	11.3	135.8
Age, *y*													
17–24	7.2	7.2	7.6	8.2	8.5	8.9	9.3 ^d^	10.1	11.9	13.3	14.6	7.4	102.3
25–34	16.3	16.3	17.0	17.9	18.5	19.1	19.6	20.5 ^d^	23.1	25.0	26.8	10.5	64.5
35–44	24.8	24.9	25.5	26.6	27.6	28.1	28.6	29.7 ^d^	31.8	33.5	35.0	10.2	41.2
45–62	22.6	22.7	23.7	24.7	26.4	27.0	27.1	28.0 ^d^	29.8	31.7	33.0	10.4	46.0
Sex, age group													
Men, 17–24	7.9	7.8	8.3	8.8	9.1	9.6	9.9 ^d^	10.7	12.6	13.9	15.1	7.3	92.7
Men, 25–34	17.5	17.5	18.2	19.1	19.6	20.2	20.7	21.5 ^d^	24.2	26.1	27.8	10.3	58.9
Men, 35–44	26.3	26.4	27.0	28.1	28.9	29.4	29.9	31.0 ^d^	33.0	34.7	36.2	9.9	37.7
Men, 45–62	24.0	24.1	25.1	26.1	27.9	28.5	28.5	29.4 ^d^	31.2	33.1	34.4	10.4	43.3
Women, 17–24	4.2	4.2	4.4	5.0	5.5	6.0	6.9 ^d^	7.7	9.4	10.9	12.5	8.3	195.2
Women, 25–34	9.7	9.7	10.3	11.4	12.3	13.3	14.4 ^d^	15.6	18.3	20.5	22.6	12.9	132.3
Women, 35–44	15.2	15.2	16.4	17.5	19.5	20.5	21.2 ^d^	22.7	25.2	27.5	29.0	13.8	90.7
Women, 45–62	14.2	14.7	15.3	15.7	17.1	17.8	18.5 ^d^	20.3	21.5	23.5	25.1	10.9	77.0
Race and ethnicity													
Hispanic	15.8	15.5	16.1	17.0	17.4	17.8	18.3 ^d^	19.5	22.0	24.2	26.0	10.2	64.5
Black, non-Hispanic	21.1	20.7	21.1	21.8	22.1	22.4	22.6 ^d^	23.4	26.0	27.6	29.0	7.9	37.6
White, non-Hispanic	12.8	12.9	13.3	13.9	14.3	14.6	15.1	15.9 ^d^	18.3	20.4	22.1	9.3	72.5
Other	12.5	12.8	13.5	14.5	15.2	16.4	16.8 ^d^	18.0	20.1	21.8	23.4	10.8	86.5
Service branch ^e^													
Army	16.9	16.4	16.9	17.1	16.8	16.5	16.4	17.4 ^d^	19.8	22.2	24.2	7.3	43.3
Navy	15.8	16.3	17.0 ^d^	18.9	20.3	21.3	22.8	23.7	25.8	27.3	29.0	13.2	83.6
Air Force	13.4	13.9	14.5	15.8	16.6	17.1	17.3	18.5 ^d^	21.5	23.2	24.7	11.4	85.0
Marine Corps	6.2	6.1	6.3	6.4	6.7	7.3 ^d^	7.9	8.8	10.1	11.5	12.6	6.5	104.8
Coast Guard	17.1	16.6	17.0 ^d^	17.7	18.3	18.8	19.8	21.8	22.4	21.1	22.5	5.4	31.8

Abbreviations: *n* , number (unweighted); *y* , years.

a Defined as body mass index greater than or equal to 30.

b Data sources Defense Medical Surveillance System (DMSS) and Military Health System Data Repository (MDR), 2013-2023.

c Standardized to sex and age composition of study's active component service member population in 2023; all other groups show crude values only.

d Location of inflection point identified by Joinpoint Regression Program.

e Other service branches (n=126,820) including Space Force, Navy afloat, National Oceanic and Atmospheric Administration, Public Health Service, Office of Secretary of Defense, other / unknown included in prevalence calculations for this table but not reported separately.

**TABLE 3.** Trends in Prevalence of Obesity ^a^ , U.S. Civilians, 2013–2023 ^b^

NHANES Survey Cycle	2013–2014			2015–2016			2017–March 2020			August 2021–August 2023		
Total, *n*	4,474			4,296			6,495			4,069		
	Weighted Prevalence (%)	95% CI Lower Limit	95% CI Upper Limit	Weighted Prevalence (%)	95% CI Lower Limit	95% CI Upper Limit	Weighted Prevalence (%)	95% CI Lower Limit	95% CI Upper Limit	Weighted Prevalence (%)	95% CI Lower Limit	95% CI Upper Limit
Total												
Standardized ^c , d^	31.3	29.2	33.6	33.7	30.5	36.9	37.8	34.7	40.9	33.0	30.1	36.0
Crude	37.2	34.5	40.0	38.6	34.9	42.4	40.9	38.1	43.8	39.8	35.6	44.2
Sex												
Men ^d^	33.5	30.1	37.0	37.4	32.3	42.7	41.4	36.7	46.3	38.6	34.7	42.7
Women	40.9	37.7	44.1	39.8	36.0	43.7	40.4	37.4	43.4	41.1	35.7	46.6
Age, *y*												
17–24	23.8	19.2	28.9	26.9	22.7	31.4	29.4	24.0	35.1	24.0	19.6	28.7
25–34	35.2	31.3	39.4	35.5	30.3	40.9	40.7	34.6	46.9	37.0	29.8	44.6
35–44	42.9	39.4	46.4	43.7	36.8	50.7	44.7	41.5	48.0	42.2	36.8	47.7
45–62 ^e^	41.2	36.0	46.4	42.3	36.9	47.9	44.2	41.0	47.4	47.2	43.6	51.0
Sex, age group, *y*												
Men, 17–24	21.1	15.3	28.0	25.2	19.2	32.1	29.9	22.6	38.0	24.5	19.2	30.6
Men, 25–34 ^d^	31.9	27.1	37.1	35.3	28.0	43.0	42.0	34.1	50.3	32.7	24.0	42.5
Men, 35–44	43.3	38.6	48.0	42.9	33.9	52.2	43.7	36.7	50.9	44.4	38.4	50.7
Men, 45–62 ^e^	35.1	29.8	40.6	40.7	34.7	47.0	45.5	39.7	51.3	45.4	40.0	50.9
Women, 17–24	26.8	22.1	31.9	28.6	22.5	35.4	28.7	22.6	35.5	23.3	17.5	30.0
Women, 25–34	38.8	33.4	44.3	35.7	31.4	40.2	39.3	31.2	47.8	41.4	33.6	49.7
Women, 35–44	42.4	38.2	46.8	44.4	35.6	53.4	45.8	41.3	50.2	39.9	32.2	48.0
Women, 45–62	46.9	40.2	53.6	43.8	36.7	51.1	43.0	38.2	47.8	49.1	45.0	53.1
Race and ethnicity												
Hispanic	42.0	37.6	46.4	46.1	41.8	50.4	43.7	40.9	46.6	42.4	34.0	51.1
Black, non-Hispanic ^e^	46.8	42.5	51.2	45.1	39.8	50.4	49.3	46.3	52.3	52.7	47.3	58.1
White, non-Hispanic	36.2	32.5	39.9	37.1	32.8	41.6	40.2	35.8	44.7	39.1	35.1	43.1
Other	21.8	16.5	27.8	26.2	17.8	36.1	30.4	25.0	36.1	28.4	20.2	37.8

Note: Percentage point change and relative percentage change not calculated for civilians due to unequal NHANES survey cycle length and spacing.

Abbreviations: *n* , number (unweighted); CI, confidence interval; *y* , years.

a Defined as body mass index greater than or equal to 30.

b Data source National Health and Nutrition Examination Survey (NHANES), 2013-2014 to Aug. 2021-Aug. 2023.

c Standardized to sex and age composition of study's active component service member population in 2023; all other groups show crude values only.

d Significant quadratic trend, *p*<0.05.

e Significant linear trend, *p*<0.05.

### Trends in male and female obesity

From 2013 through 2023, crude prevalence of obesity, in both men and women, was lower among ACSMs than civilians, although the difference narrowed over time **(Figure 1)** .

**FIGURE 1a.**
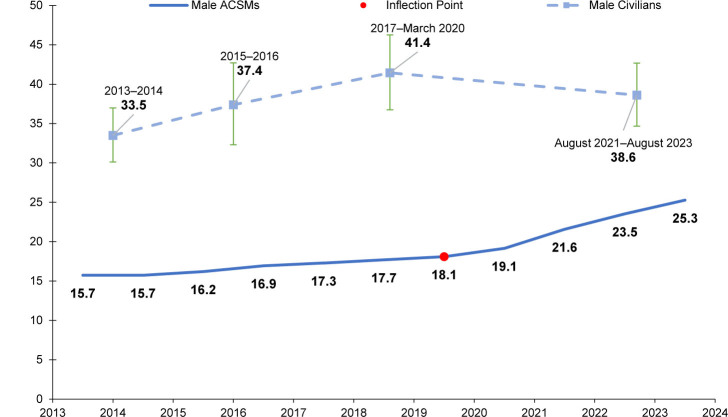
Crude Prevalence of Obesity ^a^ , Male U.S. Active Component Service Members and Civilians, 2013–2023 ^b^

**FIGURE 1b.**
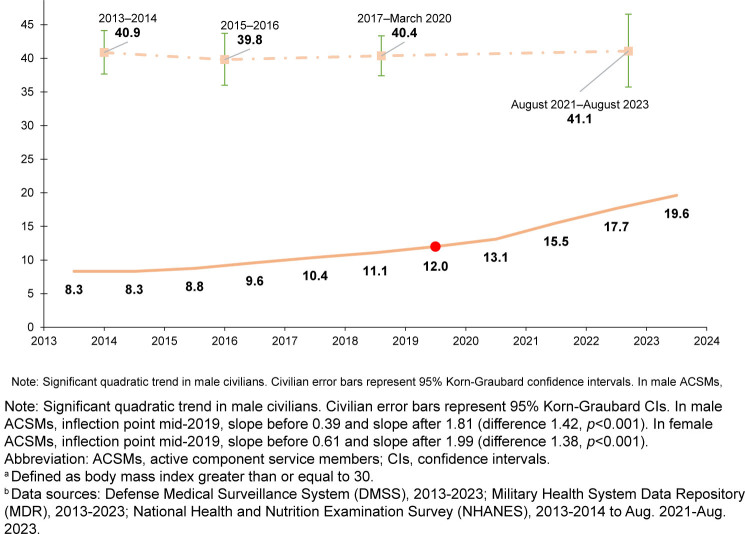
Crude Prevalence of Obesity ^a^ , Female U.S. Active Component Service Members and Civilians, 2013–2023 ^b^

Prevalence of obesity in male ACSMs increased from 15.7% in 2013 to 18.1% in 2019; from the joinpoint at 2019 obesity increased more rapidly, to 25.3% in 2023 (difference in slope before and after join-point 1.42, *p*<0.001). Obesity increased in female ACSMs from 8.3% in 2013 to 12.0% in 2019; from the joinpoint at 2019 obesity increased more rapidly, to 19.6% in 2023 (difference in slope before and after joinpoint 1.38, *p*<0.001).

Estimated prevalence of obesity in male civilians increased from 33.5% (95% CI 30.1, 37.0) in 2013-2014 to 41.4% (95% CI 36.7, 46.3) during 2017–March 2020, then declined to 38.6% (95% CI 34.7, 42.7) during August 2021–August 2023 (quadratic trend *p*=0.04, linear trend *p* =0.05). In female civilians, obesity remained consistent from 2013-2014 (40.9%; 95% CI 37.7, 44.1) to August 2021–August 2023 (41.1%; 95% CI 35.7, 46.6) (quadratic trend *p*=0.67, linear trend *p*=0.85).

### Trends in young male obesity

**FIGURE 2.**
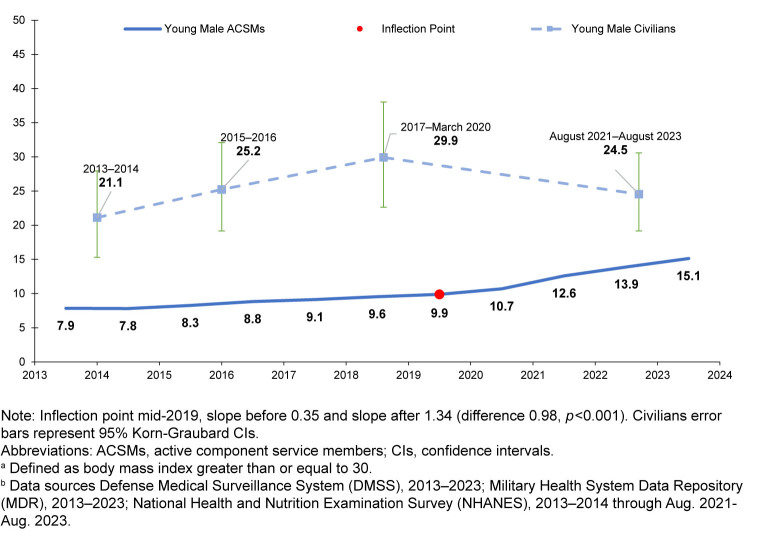
Crude Prevalence of Obesity ^a^ , Young Male (ages 17–24 years) U.S. Active Component Service Members and Civilians, 2013–2023 ^b^

Among young male (ages 17-24 years) ACSMs, crude obesity prevalence increased from 7.9% in 2013 to 9.9% in 2019; from the joinpoint at 2019, obesity increased more rapidly to 15.1% in 2023 (difference in slope before and after joinpoint 0.98, *p*<0.001) **(Figure 2)** .

Among young male civilians, estimated prevalence of obesity did not change significantly, from 21.1% (95% CI 15.3, 28.0) in 2013-2014 to 24.5% (95% CI 19.2, 30.6) during August 2021–August 2023 (quadratic trend *p* =0.08, linear trend *p* =0.35).

### Distributions of body mass index

Age-standardized distributions of BMI according to sex, among ACSMs in 2023 and civilians during August 2021–August 2023, were visibly different **(Figure 3)** . Male ACSMs demonstrated lower proportions in the highest classes of obesity (class 2 obesity 4.0%, class 3 obesity 0.7%) in comparison to the civilian male population (class 2 obesity 8.2%; 95% CI 6.5, 10.2 and class 3 obesity 5.7%; 95% CI 4.5, 7.1). This pattern was even more striking in women: Female ACSMs demonstrated even smaller proportions in the highest classes of obesity (class 2 obesity 3.6%, class 3 obesity 0.8%) when compared to women in the civilian population (class 2 obesity 9.0%; 95% CI 6.9, 11.5 and class 3 obesity 11.3%; 95% CI 9.7, 13.1).

**FIGURE 3a.**
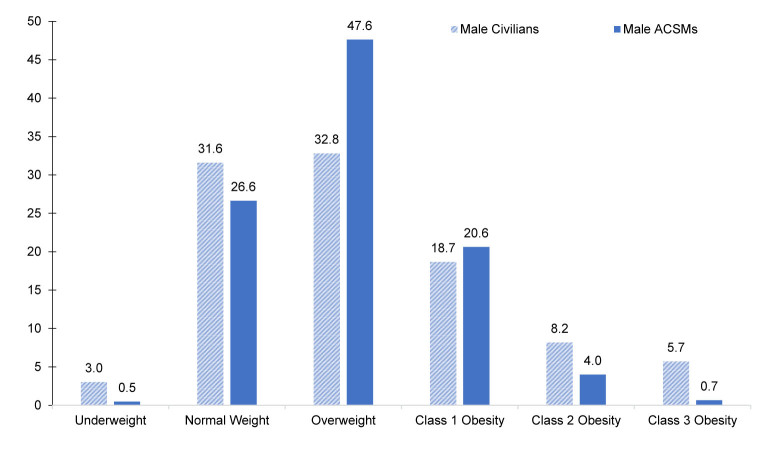
Distribution of Body Mass Index ^a^ , Male U.S. Active Component Service Members and Civilians ^b^ , 2023 and August 2021–August 2023 ^c^

**FIGURE 3b.**
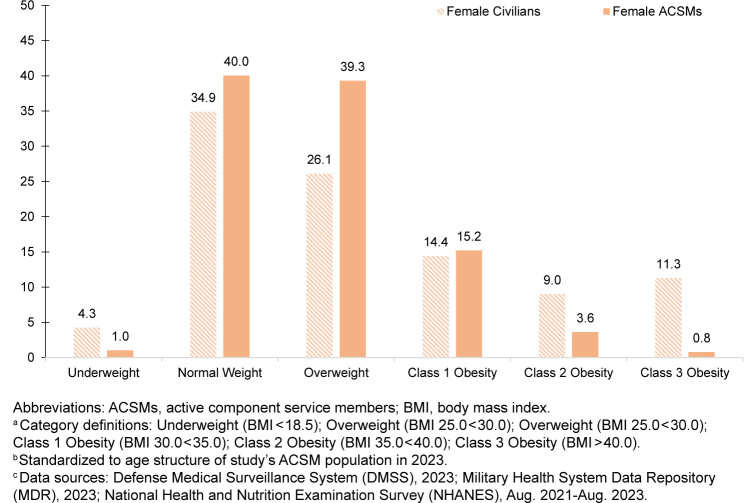
Distribution of Body Mass Index ^a^ , Female U.S. Active Component Service Members and Civilians ^b^ , 2023 and August 2021–August 2023 ^c^

## Discussion

This study included newer data, collected after the COVID-19 pandemic, to describe trends in sex- and age-standardized prevalence of obesity over the past decade among ACSMs and civilians aged 17-62 years, as well as young men.

From 2013 through 2023, the prevalence of obesity in male and female ACSMs increased, while standardized estimated prevalence among civilians ages 17-62 years increased slightly but ended similar to the start of the decade. The difference in obesity prevalence between the populations apparently narrowed. Interestingly, a majority of the increase in ACSM obesity prevalence occurred recently, from 2019 until 2023. The pattern of increasing ACSM obesity prevalence and consistently high prevalence in civilians was also present in young men (ages 17-24 years), the largest source of potential military recruits as well as newly accessed military members. More than 1 in 5 young male civilians had obesity throughout the 10-year study period.

The growing prevalence of obesity among ACSMs overall and in young men, particularly since 2019, could lead to poorer retention of newly accessed recruits and an increased burden on the MHS. The persistently high obesity prevalence among civilians presumably reduces the pool of height- and weight-eligible potential military recruits, although other factors, such as education and medical conditions, ^14^ are considered for U.S. military accession.

Force-wide changes within the DOD may explain the significantly greater 2019–2023 increase in obesity among ACSMs. In early 2020, Force Health Protection Guidance was published in response to the COVID-19 pandemic, limiting close contact and reducing workplace access, leading to suspended physical fitness testing requirements by the service branches. ^15^ This period also saw the resolution of the War on Terror and withdrawal of troops from Iraq and Afghanistan in 2021, changing the military from a wartime to peacetime posture. ^16^ These changes may have shifted emphasis from combat to non-combat occupations and reduced demand for exceptional physical capabilities.

This study has several strengths. Data collected from MDR and linked to DMSS provide a near census of ACSMs, due to the annual physical examination requirement. This study population closely matched the DOD 2023 Demographics Report. ^12^ NHANES data are nationally representative of the civilian, non-institutional population and do not rely on survey participants seeking health care. ^13^ We used measured height and weight from both data sources, which is more accurate than relying on self-reported height and weight. ^17^

This study has several limitations. Collection and interpretation of the data sources differed. DMSS provides a nearly complete, continuous census of ACSMs in MDR, while NHANES is a cross-sectional survey with a sample selected through a complex, multi-stage probability design. Statistical power to detect significant civilian trends was lower than for ACSMs due to the smaller NHANES sample size. Data from the upcoming Military Health and Nutrition Examination Study (MHANES) may be more directly comparable to NHANES. ^18^ Furthermore, the first non-pregnant record of height and weight from MDR was used for ACSMs, biasing data selection from earlier in the calendar year, whereas NHANES data are collected throughout a calendar year. Seasonal variations in body weight may occur, although the magnitude is likely small. ^19^ Additionally, it could not be ascertained whether ACSMs had obesity before joining the military or if they developed obesity after accession.

Another important consideration when interpreting the results of this study are the limitations of using BMI to define obesity. While BMI is simple, inexpensive, and widely accepted for obesity surveillance, it does not distinguish body fat from lean body mass, nor describes body fat distribution within an individual. ^20^ When comparing BMI distributions, it is apparent that a higher proportion of ACSMs than civilians are in the overweight and class 1 obesity categories; conversely, a higher proportion of civilians have class 2 and class 3 obesity (i.e., severe obesity). Some ACSM classifications of overweight or class 1 obesity are likely partially attributable to higher levels of fitness and lean muscle mass in ACSMs than in civilians. Body composition measurement has recently come under increased scrutiny and will be included in a rapid review of military standards. ^21^ Other measures of adiposity that are better proxies for central adiposity, such as waist circumference and body composition scans, may reduce some limitations of using height and weight alone to define obesity.

Future studies could compare service-specific height and weight military accession standards with the findings from this study, to ultimately inform potential effects on military strength and readiness, as well as the burden of obesity on the MHS.

## References

[1] Vergun D . DOD addresses recruiting shortfall challenges . DoD News . Dec . 13 , 2023 . Accessed May 12, 2025 . https://www.defense.gov/news/news-stories/article/article/3616786/dod-addresses-recruiting-shortfall-challenges

[2] U.S. Centers for Disease Control and Prevention . Unfit to serve. Physical Health. U.S. Dept. of Health and Human Services . Feb . 9 , 2024 . Accessed May 12, 2025 . https://www.cdc.gov/physical-activity/php/military-readiness/unfit-to-serve.html

[3] Maxey H , Bishop-Josef S , Goodman B . Un-Healthy and Unprepared: National Security Depends on Promoting Healthy Lifestyles from an Early Age . Council for a Strong America ; 2018 . Accessed May 12, 2025 . https://strongnation.s3.amazonaws.com/documents/484/389765e0-2500-49a2-9a67-5c4a090a215b.pdf

[4] Knapik JJ , Farina EK , Steelman RA , Trone DW , Lieberman HR . The medical burden of obesity and overweight in the US military: association of BMI with clinically diagnosed medical conditions in United States military service members . J Nutrition . 2023 ; 153 ( 10 ): 2951 - 2967 . doi: 10.1016/j.tjnut.2023.08.023 37619919

[5] Legg M , Stahlman S , Chauhan A , et al . Obesity prevalence among active component service members prior to and during the COVID-19 pandemic, January 2018-July 2021 . MSMR . 2022 ; 29 ( 3 ): 8 - 16 . Accessed May 12, 2025 . https://www.health.mil/news/articles/2022/03/01/obesity-prev-msmr 35485798

[6] Emmerich SD , Fryar CD , Stierman B , et al . Trends in obesity-related measures among US children, adolescents, and adults . JAMA . 2025 . doi: 10.1001/jama.2024.27676 PMC11826431 39946125

[7] Armed Forces Health Surveillance Division . Defense Medical Surveillance System. Defense Health Agency, U.S. Dept. of Defense . Updated Sep. 23, 2025 . Accessed Nov. 25, 2025 . https://www.health.mil/military-health-topics/health-readiness/afhsd/functional-information-technology-support/defense-medical-surveillance-system

[8] National Center for Health Statistics, U.S. Centers for Disease Control and Prevention . NHANES Survey Methods and Analytic Guidelines. U.S. Dept. of Health and Human Services . Accessed May 12, 2025 . https://wwwn.cdc.gov/nchs/nhanes/analyticguidelines.aspx

[9] NHLBI Obesity Education Initiative Expert Panel on the Identification, Evaluation, and Treatment of Obesity in Adults (US) . Clinical Guidelines on the Identification, Evaluation, and Treatment of Overweight and Obesity in Adults: The Evidence Report . National Heart, Lung, and Blood Institute ; 1998 . Accessed Nov. 25, 2025 . https://www.nhlbi.nih.gov/files/docs/guidelines/ob_gdlns.pdf

[10] Parker JD , Talih M , Malec DJ , et al . Vital and Health Statistics, Series 2 Number 175: National Center for Health Statistics Data Presentation Standards for Proportions–Data Evaluation and Methods Research . National Center for Health Statistics, U.S. Centers for Disease Control and Prevention ; 2017 . Accessed Nov. 25, 2025 . https://www.cdc.gov/nchs/data/series/sr_02/sr02_175.pdf

[11] Ingram DD , Malec DJ , Makuc DM , et al . Vital and Health Statistics, Series 2 Number 179: National Center for Health Statistics Guidelines for Analysis of Trends–Data Evaluation and Methods Research . National Center for Health Statistics, U.S. Centers for Disease Control and Prevention ; 2017 . Accessed Nov. 25, 2025 . https://www.cdc.gov/nchs/data/series/sr_02/sr02_179.pdf

[12] Military One Source . 2023 Demographics Profile . U.S. Dept. of Defense . 2024 . Accessed May 12, 2025 . https://www.militaryonesource.mil/data-research-and-statistics/military-community-demographics/2023-demographics-profile

[13] National Center for Health Statistics, U.S. Centers for Disease Control and Prevention . About NHANES. National Health and Nutrition Examination Survey. U.S. Dept. of Health and Human Services . Updated Dec. 18, 2024 . Accessed May 12, 2025 . https://www.cdc.gov/nchs/nhanes/about/index.html

[14] USA.gov . Requirements to Join the U.S. Military. U.S. General Services Administration . Updated Aug. 27, 2025 . Accessed May 12, 2025 . https://www.usa.gov/military-requirements

[15] Under Secretary of Defense, Personnel and Readiness . Force Health Protection (Supplement 8): DOD Guidance for Protecting Personnel in Workplaces During the Response to the Coronavirus Disease 2019 Pandemic . U.S. Department of Defense . Apr . 13 , 2020 . Accessed May 12, 2025 . https://media.defense.gov/2020/apr/13/2002280147/-1/-1/1/force-health-protection-guidance-supplement-8.pdf

[16] Council on Foreign Relations . Timeline: The U.S. War in Afghanistan . Accessed May 12, 2025 . https://www.cfr.org/timeline/us-war-afghanistan

[17] Rowland ML . Self-reported weight and height . Am J Clin Nutr . 1990 ; 52 ( 6 ): 1125 - 1133 . doi: 10.1093/ajcn/52.6.1125 2239790

[18] Berryman C , Bukhari A , Hennigar S , et al . The Military Health and Nutrition Examination Study (MHANES) project overview: assessment of the health and nutritional status of the active-duty US Army (abstract) . J Acad Nutr Diet . 2024 ; 124 ( 10 ): a79 . https://www.jandonline.org/article/s2212-2672(24)00679-8/fulltext

[19] Yanovski JA , Yanovski SZ , Sovik KN , et al . A prospective study of holiday weight gain . NEJM . 2000 ; 342 ( 12 ): 861 - 867 . doi: 10.1056/nejm200003233421206 10727591 PMC4336296

[20] Neeland IJ , Poirier P , Després JP . Cardiovascular and metabolic heterogeneity of obesity: clinical challenges and implications for management . Circulation . 2018 ; 137 ( 13 ): 1391 - 1406 . doi: 10.1161/circulationaha.117.029617 29581366 PMC5875734

[21] Secretary of Defense . Rapid Force-wide Review of Military Standards . U.S. Department of Defense . Mar . 12 , 2025 . Accessed May 12, 2025 . https://media.defense.gov/2025/mar/12/2003666182/-1/-1/1/rapid-force-wide-review-of-military-standards-osd001952-25-res-final.pdf

